# Supplementary material for: What does the demographic profile of convicts tell us about social equity in India?
Source: PLoS One. 2023 Jul 10;18(7):e0288127. doi: 10.1371/journal.pone.0288127 (PMC10332628; doi:10.1371/journal.pone.0288127)
Supplement: S1 File — (DOCX) [file pone.0288127.s001.docx]

**Supporting Information S1**

NCRB provides comprehensive statistical information on different aspects of prison administration on an annual basis. It includes data on type of prisons and prisoners, the demographic particulars of prisoners, information on Indian and foreign prisoners, distribution of convicts and undertrials offense wise, releases, deaths of prisoners, rehabilitation and welfare of prisoners among others. Even though demographic data is available at the district level, NCRB’s convict profile data is available only at the state level. Therefore, our analysis is restricted to the state-level for this reason. There is a large overlap in the demographic categories used by NCRB and Census. But there are some differences. In order to overcome these differences, we re-organised the data to make them comparable which allowed us to undertake spatial and categorical comparison. We discuss this with greater detail below.

The demographic particulars of the convicts that we have taken into account are (1) religion, (2) social stratification and (3) domicile.

1) Religion: The religious categories included in this study are: a) Hindu, b) Muslim, c) Sikh, d) Christian and e) Other religions. NCRB provides data for Hindu, Muslim, Sikh and Christian groups. The rest of the religions have been put under “Others”. To make it compatible, the census (which provides data for more religious denominations) have been grouped to match the NCRB groups.

2) Social stratification: The social stratification (caste) categories included in this study are: a) Schedule Caste (SCs), b) Schedule tribes (STs) and c) Others. We did a similar exercise, like religion, to match data categories for caste. Though the NCRB provides data for STs, SCs, OBC and Others, the census provides data for only three categories of caste, namely SCs, STs and Others. Hence for our analysis, the convict data has been organized so that the OBCs and the Others provided by the NCRB have been combined to make it comparable with the census data.

3) Domicile of origin: The domicile categories included in this study are: (a) belongs to the state, (b) belongs to other states in India and (c) belongs to other country. These are the categories used by NCRB for data on convicts. The census provides detailed information on migration by last place of residence as well as place of birth. NCRB only has data labelled as domicile but does not specify whether it is by birth or last place of residence. In order to match the two databases, we have used the place of birth data from census to match the categories of domicile from NCRB. Like religion and caste, we have matched the data categories for domicile from NCRB and census.
