# Supplementary material for: What does the demographic profile of convicts tell us about social equity in India?
Source: PLoS One. 2023 Jul 10;18(7):e0288127. doi: 10.1371/journal.pone.0288127 (PMC10332628; doi:10.1371/journal.pone.0288127)
Supplement: S2 File — (DOCX) [file pone.0288127.s002.docx]

**Supporting Information S2**

**T-test**

We use the commonly used T-test to test for equality of means between independent groups and paired samples (t-test).

Our null hypothesis is that mean (convicts) = mean(census) for all indicators (religion, caste, and domicile and their sub-categories) in each state. The t-test formula for paired sample is:

t = $\frac{difference}{(standard deviation)/square root(observations)}$ (1)

where, difference = $mean (convicts)-mean (census)$

The t-test allows us to examine whether there are significant differences in each category across states.
