# Supplementary material for: What does the demographic profile of convicts tell us about social equity in India?
Source: PLoS One. 2023 Jul 10;18(7):e0288127. doi: 10.1371/journal.pone.0288127 (PMC10332628; doi:10.1371/journal.pone.0288127)
Supplement: S3 File — (DOCX) [file pone.0288127.s003.docx]

**Supporting Information S3**

Our expectation is that a state would be socially equitous if this gap in all three categories (religion, caste and domicile) is zero.

When we calculated this gap (between prison and population proportions) we found that in most cases this gap was either negative or near zero (implying that the proportion of the dominant group in prison was less than or equal to the population) in a few cases, the gap was positive.
