# Supplementary material for: What does the demographic profile of convicts tell us about social equity in India?
Source: PLoS One. 2023 Jul 10;18(7):e0288127. doi: 10.1371/journal.pone.0288127 (PMC10332628; doi:10.1371/journal.pone.0288127)
Supplement: S4 File — (DOCX) [file pone.0288127.s004.docx]

**Supporting Information S4**

A commonly used method to avoid these problems is to use a panel data model. The panel data models are more informative, with more variations and have less collinearity among variables. Since the panel data models have greater degrees of freedom, there is an increased efficiency in estimation. However, it needs to be determined whether our model better suits the random effects (RE) or fixed effects (RE) method. In order to choose between them, we first ran the panel data model with SEI as the dependent variable and HDI/GDI as the independent variable (*xtreg* command in Stata 15.1). We then used the Hausman test to choose between the two. We found that the coefficient from RE is significantly less than FE, which yielded χ^2^ < 0. This is a problem sometimes encountered in empirical analysis and the proposed solution is to use the absolute values of the coefficients to determine if these are significantly different. Since the two coefficients were significantly different in absolute value, we chose the FE model over the RE model.

To consider the neighbourhood effect, the spatial regression models consider three types of interaction effects- a) Endogenous – when the dependent variable in one region is correlated to the dependent variable in the neighbouring regions b) Exogenous-when the dependent variable of one region is correlated to the independent variable of the neighbouring region and c) interaction among the error terms of the model.
