# Supplementary material for: What does the demographic profile of convicts tell us about social equity in India?
Source: PLoS One. 2023 Jul 10;18(7):e0288127. doi: 10.1371/journal.pone.0288127 (PMC10332628; doi:10.1371/journal.pone.0288127)
Supplement: S5 File — (DOCX) [file pone.0288127.s005.docx]

**Supporting Information S5**

The density of distribution “ϕ_t_” is expected to evolve in accordance with the following equation (8):

ϕ _t_ = M. ϕ _t-1_ (8)

where, “M” maps the transition in the SEI for two consecutive periods “t” and “t-1”. As a first-order Markov process, the density distribution “ϕ” for the period “t” only depends on the density “ϕ” for the immediately preceding period “t-1”. In our estimates below we have assumed that the distribution “ϕ” has a finite number of states According to the first-order Markov chain, the probability of variable s_t_ taking a particular value depends only on its past value s_t-1_ as stated in equation (9) below:

*P{s _t_ = j │ s _t-1_ = i }=*  *P_i j_* (9)

where P_ij_ indicates the probability that state i will be followed by state j.

By observing the transitions out of or into the discrete cells, we learn how many transitions are taking place in the given time period. In our analysis of SEI transition, the location of each state in terms of SEI band position is first identified. We then examine which states remained in the same band in the initial period and in the terminal period. A transition to another band indicates mobility, while being in the same band indicates persistence. A transition to above or below the diagonal indicates a change in relative performance.
