# Supplementary material for: What does the demographic profile of convicts tell us about social equity in India?
Source: PLoS One. 2023 Jul 10;18(7):e0288127. doi: 10.1371/journal.pone.0288127 (PMC10332628; doi:10.1371/journal.pone.0288127)
Supplement: S6 File — Summary statistics of average crime rates by different category of states. (DOCX) [file pone.0288127.s006.docx]

**Supporting Information S6**

**Table S6. Summary statistics of Average crime rates by different category of states**

|  | Crime Rate (Average) | | |  |
| --- | --- | --- | --- | --- |
| Year | EAG | Non EAG | Special | General |
| 2001 | 158.88 | 187.96 | 140.49 | 196.48 |
| 2011 | 172.06 | 196.88 | 140.07 | 210.67 |

Source: Authors’ calculations based on NCRB data.
