# Supplementary material for: What does the demographic profile of convicts tell us about social equity in India?
Source: PLoS One. 2023 Jul 10;18(7):e0288127. doi: 10.1371/journal.pone.0288127 (PMC10332628; doi:10.1371/journal.pone.0288127)
Supplement: Attachment — Submitted filename: review.pdf [file pone.0288127.s007.pdf]

Thank you for the opportunity to review this paper. The authors used secondary data from India to research aspects of social equity nationally by comparing demographics of prisoners currently serving sentences compared to demographic characteristics. The authors use secondary survey data to quantitatively analyse the research question of whether religion, caste and domicile of prisoners differ from the characteristics of non-prison population instates. Spatial panel analysis as well as distribution dynamics approach is used to assess bias and transition across time, from 2001 to 2011. The authors report that social equity is higher in low developmental states in India, traditionally called BIMARU states, as opposed to states with better social demographic and developmental indicators. The implications of this paper and research findings were not adequately articulated.

On the whole, the paper presents novel findings using a method that constructs an index as a measure of social equity to comment on social equity in the country by measuring the composition of prisoners.

I am sharing my review, which comprises of queries and suggestions to assist the authors to revise the current version of the paper for potential publication.

Comment

In the introduction section, the authors provide global contexts with racial inequalities in the United States and Europe as a precursor to explain social inequalities where racial factors predominate. The transition to India and social inequality in India is not adequately linked with the global context. The colonial history of Europe and the Americas differs from India's history and the authors would be better suited to contextualise the research for India either for similar South Asian countries or even focusing on India alone in the introduction.

Questions

Lines 84 to 94, the authors rationalise that the law enforcement system in India is expected to be unbiased as a reflection of social equity and postulate that a difference in the proportion of prisoners and their proportion in the population would help draw out social inequities. This hypothesis is concerning as historical data from India identifies that caste-based discrimination has led to two wide social inequality and religion has been recognised as a linking factor for social deprivation, especially with Muslims. Thus the central hypothesis in the paper in lines 84 to 87 needs to be re-articulated against existing literature from India. It is

valid to postulate for some states in India, especially those with low social demographic and developmental indicators, that prisoners would be more likely to belong to certain castes religion, and that they would not be different from the majority population of that state.

Comment:

In the methodology, the authors reflect the weaknesses broadly in regression methods; however, they do not explain why regression was not an optimal method to answer their research question.

Query

The use of the social equity index is described as a methodological innovation; however, it is common in the social sciences to use conceptual frameworks and indexes as done in this paper to answer a research question. The authors may like to explain why their index should not be better classified as a conceptual innovation as compared to a methodological innovation

Question;

The presentation of the method section is slightly problematic as the various methods are drawn do not adequately explain how individual research aims are answered. The authors may like to add the research aims in the methods section and perhaps link individual methods to answer specific research questions
