# Supplementary material for: What does the demographic profile of convicts tell us about social equity in India?
Source: PLoS One. 2023 Jul 10;18(7):e0288127. doi: 10.1371/journal.pone.0288127 (PMC10332628; doi:10.1371/journal.pone.0288127)
Supplement: Attachment — Submitted filename: PONE-D-21-38262R1-Mukhopadhyay et al-Academic Editors comments.docx [file pone.0288127.s009.docx]

PONE-D-21-38262R1

What does the demographic profile of convicts tell us about social equity in India?

Pranab Mukhopadhyay ^1^*, Shaila Desouza^2^ , Aparna P. Lolayekar^2^

Comments from the Academic Editor

Line 33 SEI. Is this a composite index? If so, what are the components of the index (such as index of caste, index of religion and index of domicile)?

Lines 39-42; 181; 290; 292; 294;446; 462; 486; 489; 574-576; 581

The acronym “BIMARU” (standing for Bihar, Madhya Pradesh, Rajasthan and Uttar Pradesh) was coined by the late demographer Ashish Bose in the 1980s to highlight the lack of progress of these states in family planning, control of population growth and general socio-economic development. The acronym appeared to be apt at the time, it was first used because the word *bimaru* in Hindi also means sickly, although its use was not without criticism. However, this acronym appears not to be in vogue anymore. Therefore, please remove the acronym BIMARU from the manuscript and use the more recent terminology EAG (Empowered Action Group) states, which comprise the eight states of Bihar, Chattisgarh, Jharkhand, Madhya Pradesh, Orissa, Rajasthan, Uttaranchal, and Uttar Pradesh.

(<https://www.careers360.com/eag-states-full-form>)

Lines 117-123. Discrimination on the basis of religion may also be confounded by socio-economic status. A religious group which is discriminated against may be poorer in literacy/education and economic status. Do you acknowledge this?

Lines 163-165. Were the convicts you have studied all males, or were there females included?

Are discriminations based on caste, religion and domicile the same for men and women? Were the convicts you have studied all males, or were there females included?

Lines 166-167, Question b). This question needs clarification/elaboration. Are you looking at differentials in social equity by state or are you looking at differentials in social equity within states?

Line 177. It is not clear what you mean by “dominant” group. Dominant in what sense? In fact, the entire sentence needs clarification.

Lines 178-180. Both the HDI and GDI are available in “inequality-adjusted” form. But these indices comprise scores on life expectancy, education and income, and not religion, caste or domicile.

One can argue that indices like HDI or GDI (or their inequality-adjusted forms) are in fact influenced by inequalities among religion, caste and domicile.

Lines 178-179. Please explain how, because your SEI is based on different characteristics.

It is difficult to see how HDI and GDI would have any impact on the newly constructed SEI, or *vice versa.*

Lines 180-181. Are the overall crime rates of the BIMARU/EAG states higher, or lower than those of the non-BIMARU/EAG states?

Lines 199-202. Data that are in public domain also need Ethics clearance in the first place to collect (example, Demographic and Health Survey data). Are you aware of any ethics clearance for the data you have used and state what kind of ethic clearance(s) have been obtained?

Lines 203-204. The use of different datasets is common in many studies with each study using its own combination of data sets, but it cannot be called an “innovation”! An innovation may be in an innovative data collection or in the methodology.

Line 211. The first question examines social equity **in the law enforcement system** of each state.

Line 213. Please correct to: Spatial dimension to social equity **in each state**.

Line 214. Please correct to: Change in social equity **among the states**.

Line 218. “overcomes” or does not require?

Line 230. What is the dominant” group?

Line230. The formula, as it is worded does not take the difference (gap) between similar things of two groups.

The wording of first expression of this definition is misleading, as it would imply proportions of convicts (and non-convicts in the dominant group).

Do you mean “proportion of the dominant group among the convicts”? Please check that you have used the correct proportion in the first expression of the right-hand side of the formula.

Line 231. What is the “dominant” group?

Lines 239-240. This appears to be correct. Please modify the wording of your Equation 1 accordingly.

Line 240. What is the “dominant” group?

Line 242. Do you mean the gap, as purported to be shown in Equation 1?

Lines 244-246. The “Others” category is an amorphous or undefined group. How can any conclusion or policy recommendation be made for an undefined group? I suggest you consider the next proportionately largest group in this instance.

Lines 246-248. Have you considered the possibility that the group “born in the state” could comprise a mixture of religions and castes? What would that imply for your results and conclusions?

Lines 256-266. Please elaborate how Rel Gap_it_, Rel min_it_ etc are calculated. What have you assumed for Rel max _it_, and why? Please elaborate the same also for caste and domicile.

In calculating the various indices (or scores) for HDI standard maximum and minimum values of life expectancy, education and per capita income are taken. What maxima and minima have you assumed for the gaps in religion, caste and domicile and why have you assumed them so?

Lines 268-272. The United Nations assigns equal weights to each of the three indices of human development with the implicit assumption that each of them contributes equally to HDI. How true is that for the three indices of social equity in your study? There needs to be a discussion about assigning equal weights to the indices of caste, religion and domicile. Further, as I have mentioned earlier, domicile is an amorphous indicator in which caste and religion may be mixed up. This raises a further question similar to that of multicollinearity as in multiple regression analysis. Please provide a satisfactory discussion on this matter.

Lines 283-286. These are repetitions of the previous lines.

Lines 296-298. Please state briefly what the limitations are.

Lines 520-521. Centrally appointed administrators like the IAS, IPS are often posted in states other than the state of their own birth/domicile. As such, they would be much less likely to be influenced to grant favours to alleged criminals from the state (domicile) where these administrators are posted. Are there elements of political intervention?

Line 571. The acronym BIMARU was a convenient way to describe the states which lagged behind other states in family planning and fertility decline and not exactly in terms of human development.

Lines 568 and onwards. Please see my previous comments about using BIMARU and replacing it with EAG states.
